# Supplementary material for: Evaluating indirect genetic effects of siblings using singletons
Source: PLoS Genet. 2022 Jul 7;18(7):e1010247. doi: 10.1371/journal.pgen.1010247 (PMC9262210; doi:10.1371/journal.pgen.1010247)
Supplement: S1 Text — Table A in S1 Text. PGS association estimates for singletons and non-singletons. Table displays association estimates between height, BMI and educational attainment PGS with the same phenotype in singletons and non-singletons. Table B in S1 Text. PGS association estimates for firstborns and non-firstborns. Table displays association estimates between height, BMI and educational attainment PGS with the same phenotype in firstborns and non-firstborns. Table C in S1 Text. Characteristics of singletons, non-singletons, firstborns and non-firstborns in UK Biobank. Table contains descriptives of group-level characteristics of singletons, non-singletons, firstborns and non-firstborns. Table D in S1 Text. Differences between singletons and non-singletons. Table contains estimates of differences in group-level characteristics between singletons and non-singletons. Table E in S1 Text. Differences between singletons and firstborns. Table contains estimates of differences in group-level characteristics between firstborns and non-firstborns. (DOCX) [file pgen.1010247.s001.docx]

**Supplementary Materials**

Derivation of ${Cov}_{NS}[G_{I}, Y]$

We define a phenotype $Y$ as a function of the index individual ($G_{I}$), maternal ($G_{M}$), paternal ($G_{P}$) and sibling ($G_{S}$) genotypes.

$$Y = k_{I}G_{I} + k_{M}G_{M}+k_{P}G_{P}+k_{S}G_{S}+\in$$

where$k_{I,M,P,S}$are the (direct/indirect) effects of $G_{I,M,P,S}$ on $Y$

Our aim is to derive the covariance between $G_{I}$ and $Y$ in singletons ${(Cov}_{S}[G_{I}, Y] )$and non-singletons ${(Cov}_{NS}[G_{I}, Y] )$. Here, we derive ${Cov}_{NS}[G_{I}, Y]$.

First, we consider the general derivation for covariance in terms of expectations.

(1) $Cov[X, Y] = E[XY] - (E[X]E[Y])$

From (1) we note that:

$${Cov}_{NS}[G_{I}, Y] = E[G_{I}Y] - (E[G_{I}]E[Y])$$

We can expand $E\left[ G_{I}Y \right]$ as follows:

$$E\left[ G_{I}Y \right]= E\left[ k_{I}{G_{I}}^{2}+k_{M}G_{M}G_{I}+k_{P}G_{P}G_{I}+k_{S}G_{S}G_{I} \right]$$

$$= k_{I}E[{G_{I}}^{2}] + k_{M}E\left[ G_{M}G_{I} \right]+k_{P}E\left[ G_{P}G_{I} \right]+k_{S}E[G_{S}G_{I}]$$

From (1) it follows that:

$E[{G_{I}}^{2}]=E$[$G_{I}]^{2}+{Cov}_{NS}[G_{I}, G_{I}]$

$$=E[G_{I}]^{2} + Var[G_{I}]$$

From (1) and the correlation of 0.5 between $G_{I}$ and $G_{M,P,S}$ it follows that:

$E[G_{M}G_{I}$] $=E\left[ G_{P}G_{I} \right]=E\left[ G_{S}G_{I} \right]$

$$= E\left[ G_{M} \right]E\left[ G_{I} \right]+{Cov}_{NS}[G_{M}, G_{I}]$$

$$=E[G_{I}]^{2} + 0.5Var[G_{I}]$$

Therefore, we can simplify $E\left[ G_{I}Y \right]$ to the following:

(2) $E\left[ G_{I}Y \right]$ =$\left( k_{I}{+0.5k}_{M}+{0.5k}_{P}+{0.5k}_{S} \right)Var[G_{I}]$ + $\left( k_{I}{+k}_{M}+k_{P}+k_{S} \right)E[G_{I}]^{2}$

We can derive $E\left[ Y \right]$ as a function of $k_{I,M,P,S}$ and ${E[G}_{1}]$:

$$E\left[ Y \right]=\left( k_{I}+k_{M}+k_{P}+k_{S} \right)E[G_{I}]$$

It then follows that:

$$\left( 3 \right)E[G_{I}]E[Y])=\left( k_{I}+k_{M}+k_{P}+k_{S} \right)E[G_{I}]^{2}$$

Finally, from (1), (2) and (3) we can simplify ${Cov}_{NS}\left[ G_{I}, Y \right]$ to:

$${Cov}_{NS}\left[ G_{I}, Y \right]= \left( k_{I}+{0.5k}_{M}+{0.5k}_{P}+{0.5k}_{S} \right)Var[G_{I}]$$

Comparison of Howe et al sibling IGE estimates with Kong et al

In this manuscript, we report evidence of sibling IGEs using singleton status data in unrelated individuals. In a recent preprint, Kong et al ^1^ reported limited evidence of sibling IGEs using a family-based approach.

To evaluate whether differences in conclusions relate to statistical power or to genuine heterogeneity, we compared the estimates. The two analyses used different (overlapping) datasets from the UK Biobank and distinct methodologies. For example, Kong et al ^1^ used an educational attainment PGS including a larger number of variants (and so is more predictive) than the PGS that we used, suggesting that caution is required when interpreting a direct comparison.

Kong et al ^1^ estimated that a SD increase in the sibling’s educational attainment PGS corresponds to a -0.001 SD unit change in the index individual’s educational attainment (95% C.I. -0.026, 0.024). We estimated that a SD increase in a sibling’s PGS corresponds to a 0.025 (0.013, 0.036) year increase in the index individual’s educational attainment.

Kong et al ^1^ presented their estimates in standard deviation units while we present our estimates in years in educational attainment. Therefore, considering that the standard deviation of educational attainment is 2.3 years (after adjusting for sex and birth year), we rescaled the Kong et al ^1^ estimates to be expressed in terms of years of educational attainment and so more directly comparable to our estimates. The scaled Kong et al ^1^ estimate is that a SD increase in the sibling’s educational attainment PGS corresponds to a -0.0023 year (95% C.I. -0.061, 0.057) change in the index individual’s educational attainment. We also scaled the overall PGS association estimate from Kong et al ^1^ to years of education and compared the ratio of the sibling IGE estimates over the overall PGS association.

|  | Howe et al  *Estimate (95% C.I.)* | Kong et al ^1^  *Estimate (95% C.I.)* |
| --- | --- | --- |
| Sibling PGS to index individual educational attainment  (years of schooling) | 0.025 (0.013, 0.036) | -0.002 (-0.061, 0.057) |
| Index PGS to index individual educational attainment  (years of schooling) | 0.23 | 0.55 |
| Sibling PGS association divided by index PGS association  (%) | 11% (6%, 16%) | 0% (-11%, 10%) |

There was limited statistical evidence of heterogeneity between the sibling IGE estimates from the two studies. The ratios of the sibling PGS estimate over the overall PGS association estimate were also broadly consistent between the two studies. This suggests that differences in statistical power are likely to explain the differences in conclusions between our work and that of Kong et al ^1^.

**Supplementary Tables**

**Table A** PGS association estimates for singletons and non-singletons.

| **Outcome** | **Non-singletons**  **(N = 328,549)** | **Singletons**  **(N = 50,143)** | **Singleton difference % (95% C.I.)** | **Heterogeneity P-value** |
| --- | --- | --- | --- | --- |
| Height:  Increase (cm) per SD increase in height PGS | 2.32 (2.30, 2.34) | 2.32 (2.27, 2.38) | 0% (-3%, 2%) | 0.86 |
| BMI:  Increase (kg/m2) per SD increase in BMI PGS | 0.73 (0.72, 0.75) | 0.70 (0.66, 0.74) | 5% (-1%, 11%) | 0.13 |
| Education:  Increase (years) per SD increase in education PGS | 0.24 (0.23, 0.25) | 0.21 (0.19, 0.23) | 12% (3%, 22%) | 0.009 |
| Education analysis  using within-sibship GWAS weights | 0.18 (0.17, 0.18) | 0.16 (0.14, 0.18) | 11% (-1%, 23%) | 0.08 |

**Table B** PGS association estimates for firstborns and non-firstborns.

| **Outcome** | **Firstborn**  **(N = 43,733)** | **Non-firstborn**  **(N = 66,593)** | **Heterogeneity P-value** |
| --- | --- | --- | --- |
| Height:  Increase (cm) per SD increase in height PGS | 2.36 (2.30, 2.41) | 2.28 (2.24, 2.33) | 0.04 |
| BMI:  Increase (kg/m2) per SD increase in BMI PGS | 0.72 (0.68, 0.77) | 0.71 (0.67, 0.74) | 0.55 |
| Education:  Increase (years) per SD increase in education PGS | 0.21 (0.19, 0.23) | 0.24 (0.23, 0.26) | 0.013 |

**Table C** Characteristics of singletons, non-singletons, firstborns and non-firstborns in UK Biobank.

| **Phenotype** | **Singletons**  **(N = 50,143)** | **Non-singletons**  **(N = 328,549)** | **Firstborn**  **(N = 43,733)** | **Non firstborn**  **(N = 66,593)** |
| --- | --- | --- | --- | --- |
| Sex:  % male | 46.8% | 45.8% | 46.6% | 45.3% |
| Birth year:  Mean (SD) | 1949 (7.4) | 1952 (8.1) | 1952 (8.2) | 1953 (8.1) |
| North-South birth coordinates (km north) | 353.0 (157.4) | 358.2 (164.6) | 319.9 (142.4) | 325.7 (139.6) |
| East-West birth coordinates (km east) | 416.6 (78.1) | 413.1 (81.1) | 433.4 (79.1) | 431.0 (77.2) |
| Height (cm):  Mean (SD) | 168.7 (9.3) | 168.8 (9.2) | 169.3 (9.2) | 168.8 (9.2) |
| BMI (kg/m^2^):  Mean (SD) | 27.5 (4.8) | 27.3 (4.8) | 27.4 (4.8) | 27.3 (4.7) |
| Education (years):  Mean (SD) | 14.3 (2.3) | 14.2 (2.3) | 14.5 (2.3) | 14.1 (2.3) |

**Table D** Differences between singletons and non-singletons.

| **Phenotype** | **Singleton minus non-singleton: Difference (95% C.I.)** |
| --- | --- |
| Sex:  % male | 1.0% (0.6%, 1.5%) |
| Birth year:  Mean (SD) | 2.6 (2.6, 2.7) |
| North-South birth coordinates (km north) | -5.2 (-3.6, -6.8) |
| East-West birth coordinates (km east) | 3.5 (2.7, 4.3) |
| Height (cm)*:  Mean (SD) | 0.15 (0.09, 0.21) |
| BMI (kg/m^2^)*:  Mean (SD) | 0.06 (0.01, 0.10) |
| Education (years)*:  Mean (SD) | 0.25 (0.23, 0.27) |
| Educational attainment standardised** PGS:  Mean (SD) | -0.002 (-0.011, 0.008) |

* adjusted for sex and birth year .

** to mean of 0 and standard deviation of 1.

**Table E** Differences between singletons and firstborns.

| **Phenotype** | **Singleton minus firstborn **:**  **Difference (95% C.I.)** |
| --- | --- |
| Sex:  % male | 0.2% (-0.4%, 0.9%) |
| Birth year:  Mean (SD) | 2.9 (2.8, 3.0) |
| North-South birth coordinates (km north) | 33.1 (31.1, 35.1) |
| East-West birth coordinates (km east) | -6.8 (-15.8, -17.9) |
| Height (cm)*:  Mean (SD) | -0.23 (-0.31, -0.14) |
| BMI (kg/m^2^)*:  Mean (SD) | 0.08 (0.02, 0.15) |
| Education (years)*:  Mean (SD) | -0.09 (-0.12, -0.06) |
| Educational attainment standardised*** PGS:  Mean (SD) | -0.012 (-0.024, 0.001) |

* adjusted for sex and birth-year.

** firstborns are non-singletons with only younger siblings.

*** to mean of 0 and standard deviation of 1.

References

1. Kong A, Benonisdottir S, Young AI. Family Analysis with Mendelian Imputations. biorXiv. 2020:2020.07.02.185181. doi: 10.1101/2020.07.02.185181 %J bioRxiv.
