# Supplementary material for: Evaluating indirect genetic effects of siblings using singletons
Source: PLoS Genet. 2022 Jul 7;18(7):e1010247. doi: 10.1371/journal.pgen.1010247 (PMC9262210; doi:10.1371/journal.pgen.1010247)
Supplement: Attachment — Submitted filename: PLOSgeneticsD2100943Review.docx [file pgen.1010247.s002.docx]

Manuscript Number: PGENETICS-D-21-00943

Title: Evaluating indirect genetic effects of siblings using singletons

Authors: Laurence J Howe, David M Evans, Gibran Hemani, George Davey Smith, and Neil M Davies

Howe et al have written an excellent article that extends the uses of PGS to estimate the indirect genetic effects of siblings’ and parents’ genotypes on complex traits. The approach relies on a very simple but elegant design, comparison of the PGS coefficient values for a complex trait using cohorts of individuals with and without siblings. Variations on the approach can include the number of siblings and birth order. Their design complements designs that require explicitly recruiting and measuring family members.

I have only one suggestion and one minor comment:

Suggestion: As with any epidemiological study, the interpretation of the inference depends on how well the data conform to the modeling assumptions and how robust the model is to violation of those assumptions. To their credit, the authors state that there are alternative explanations, they include some comparisons of group-level differences, and they discuss the qualitative effects of violating the assumptions (methods: page 13), however they do not include any theoretical/simulation studies that provide quantitative estimates of the degree to which their models are sensitive to their assumptions. This is particularly important because, as they show in their real data examples, the indirect sibling effect can be rather small albeit statistically significant. It would be very helpful to have some analytical or simulation results that quantify the effects of model misspecification on the estimates. As examples: How inconsistent do the other sources of genetic association (between the singleton and nonsingleton cohorts) need to be for the model to infer an indirect sibling effect that is not actually present? How much would confounding factors between parental and sibling genotypes distort the results?

Minor comment: Page 12, methods: The derivation of Cov_ns(G_i,Y) seems to assume that there are only additive (independent allelic) genetic effects. Although this assumption is reasonable, it should be explicitly stated.
