# Supplementary material for: Evaluating indirect genetic effects of siblings using singletons
Source: PLoS Genet. 2022 Jul 7;18(7):e1010247. doi: 10.1371/journal.pgen.1010247 (PMC9262210; doi:10.1371/journal.pgen.1010247)
Supplement: Attachment — Submitted filename: PLOSGenetics_Response_Dec2021.docx [file pgen.1010247.s003.docx]

Reviewer #1:

Howe et al have written an excellent article that extends the uses of PGS to estimate the indirect genetic effects of siblings’ and parents’ genotypes on complex traits. The approach relies on a very simple but elegant design, comparison of the PGS coefficient values for a complex trait using cohorts of individuals with and without siblings. Variations on the approach can include the number of siblings and birth order. Their design complements designs that require explicitly recruiting and measuring family members.

I have only one suggestion and one minor comment:

Suggestion: As with any epidemiological study, the interpretation of the inference depends on how well the data conform to the modeling assumptions and how robust the model is to violation of those assumptions. To their credit, the authors state that there are alternative explanations, they include some comparisons of group-level differences, and they discuss the qualitative effects of violating the assumptions (methods: page 13), however they do not include any theoretical/simulation studies that provide quantitative estimates of the degree to which their models are sensitive to their assumptions. This is particularly important because, as they show in their real data examples, the indirect sibling effect can be rather small albeit statistically significant. It would be very helpful to have some analytical or simulation results that quantify the effects of model misspecification on the estimates. As examples: How inconsistent do the other sources of genetic association (between the singleton and nonsingleton cohorts) need to be for the model to infer an indirect sibling effect that is not actually present? How much would confounding factors between parental and sibling genotypes distort the results?

We thank the reviewer for their kind feedback on the manuscript and their helpful suggestion.

We have added to the assumption section (below in bold) the expected bias if each assumption does not hold.

“Assumption 1: No heterogeneity in direct effects ($k_{I}$) between non-singletons and singletons.

$$k_{NS,I}=k_{S,I}$$

**If this assumption does not hold then the difference estimator will be biased by the difference in direct effects** $\boldsymbol{(}\boldsymbol{k}_{\boldsymbol{NS,I}}\boldsymbol{-}\boldsymbol{k}_{\boldsymbol{S,I}}\boldsymbol{)}$**.**

$${\hat{\boldsymbol{\beta}}}_{\boldsymbol{NS}}\boldsymbol{-}{\hat{\boldsymbol{\beta}}}_{\boldsymbol{S}}\boldsymbol{=}\boldsymbol{k}_{\boldsymbol{S}}\boldsymbol{+(}\boldsymbol{k}_{\boldsymbol{NS,I}}\boldsymbol{-}\boldsymbol{k}_{\boldsymbol{S,I}}\boldsymbol{)}$$

Assumption 2: No heterogeneity in maternal ($k_{M}$) or paternal ($k_{P}$) indirect effects between non-singletons and singletons.

$$k_{NS,M}=k_{S,M}$$

$$k_{NS,P}=k_{S,P}$$

**If this assumption does not hold then the difference estimator will be biased by half of the differences in maternal and paternal indirect effects** $\boldsymbol{(}\boldsymbol{k}_{\boldsymbol{NS,M}}\boldsymbol{-}\boldsymbol{k}_{\boldsymbol{S,M}}\boldsymbol{,}\boldsymbol{k}_{\boldsymbol{NS,P}}\boldsymbol{-}\boldsymbol{k}_{\boldsymbol{S,P}}\boldsymbol{)}$**.**

$${\hat{\boldsymbol{\beta}}}_{\boldsymbol{NS}}\boldsymbol{-}{\hat{\boldsymbol{\beta}}}_{\boldsymbol{S}}\boldsymbol{=}\boldsymbol{k}_{\boldsymbol{S}}\boldsymbol{+0.5}\left( \boldsymbol{k}_{\boldsymbol{NS,M}}\boldsymbol{-}\boldsymbol{k}_{\boldsymbol{S,M}} \right)\boldsymbol{+0.5(}\boldsymbol{k}_{\boldsymbol{NS,P}}\boldsymbol{-}\boldsymbol{k}_{\boldsymbol{S,P}}\boldsymbol{)}$$

Assumption 3: No heterogeneity in confounders of $G_{I}$ and $Y$ between non-singletons and singletons.

**If this assumption does not hold then the difference estimator will be biased by the confounder** $\boldsymbol{C}$ **which must influence both** $\boldsymbol{G}_{\boldsymbol{I}}$ **and** $\boldsymbol{Y}$ **by definition so must be a function of** $\boldsymbol{G}_{\boldsymbol{I}}$ **in the context of** $\boldsymbol{Y}$**.**

$${\hat{\boldsymbol{\beta}}}_{\boldsymbol{NS}}\boldsymbol{-}{\hat{\boldsymbol{\beta}}}_{\boldsymbol{S}}\boldsymbol{=}\boldsymbol{k}_{\boldsymbol{S}}\boldsymbol{+}\boldsymbol{C}^{\boldsymbol{'}}$$

**where** $\boldsymbol{C}^{\boldsymbol{'}}$ **is a derivative of** $\boldsymbol{C}$**.**

We have also added simulations to the methods to confirm the theory and to investigate the impact of assortative mating on the framework. Simulations confirmed the theoretical expectations and illustrated that assortative mating will likely lead to upward bias when estimating sibling indirect genetic effects.

“To explore properties of the proposed framework we performed simulations. First, we confirmed the theory from above that sibling indirect genetic effects will lead to differences in genetic associations between singletons and non-singletons in simulated data. Second, we evaluated how non-random mating affects parameter estimates from the framework. Code for simulations is available at [SiblingIGE/simulations.R at main · LaurenceHowe/SiblingIGE (github.com)](https://github.com/LaurenceHowe/SiblingIGE/blob/main/simulations.R).

Model 1 – Random mating

In Model 1, we simulated 100,000 parent-offspring trios with singleton offspring and 100,000 parent-offspring quads with two non-singleton offspring (i.e. a sibling pair).

a) Parent-offspring trios

We simulated a normally distributed PGS for each individual using the following variance-covariance matrix to characterise within-family correlations for maternal $({PGS}_{M}$), paternal $({PGS}_{P}$), and offspring (${PGS}_{O}$) PGS under random mating.

$$M P O$$

$$\begin{matrix} 1 & 0 & 0.5 \\ 0 & 1 & 0.5 \\ 0.5 & 0.5 & 1 \end{matrix}$$

The offspring phenotype $Y_{O}$was simulated as a function of the direct effect of ${PGS}_{O}$, indirect effects of ${PGS}_{M}$ and ${PGS}_{P}$, effects of confounding $C$ and a normally distributed error term ($\in\sim N\left( 0, 1 \right))$.

$$Y_{O} = k_{O}{PGS}_{O} + k_{M}{PGS}_{M}+{k_{P}PGS}_{P}+\left( C{*PGS}_{O} \right)+ \in$$

where$k_{O,M,P}$are the (direct/indirect) effects of ${PGS}_{O,M,P}$ on $Y_{O}$.

b) Parent-offspring quads

We simulated a normally distributed PGS as above for the parent-offspring trios but with two offspring using the following variance-covariance matrix.

$$M P O_{1} O_{2}$$

$\begin{matrix} 1 & 0 \\ 0 & 1 \end{matrix}$ $\begin{matrix} 0.5 & 0.5 \\ 0.5 & 0.5 \end{matrix}$

$\begin{matrix} 0.5 & 0.5 \\ 0.5 & 0.5 \end{matrix}$ $\begin{matrix} 1 & 0.5 \\ 0.5 & 1 \end{matrix}$

The phenotype of $O_{1} {(Y}_{O_{1}})$was simulated as above but including an additional effect of the PGS of their sibling $O_{2}$.

$$Y_{O_{1}} = k_{O_{1}}{PGS}_{O_{1}} + k_{M}{PGS}_{M}+{k_{P}PGS}_{P}+k_{O_{2}}{PGS}_{O_{2}}+C\left( {PGS}_{O_{1}} \right)+ \in$$

where$k_{O_{1},M,P,O_{2}}$are the (direct/indirect) effects of ${PGS}_{O_{1},M,P,O_{2}}$ on $Y_{O_{1}}$.

We then computed the OLS regression estimates of an individual’s own PGS on their phenotype, i.e., ${PGS}_{O}$ on $Y_{O}$ in the singleton sample and ${PGS}_{O_{1}}$ on $Y_{O_{1}}$ in the non-singleton sample.

Simulations confirmed the theoretical expectations from above that the OLS estimates in the singleton $\hat{\beta}_{S}$ and non-singleton $\hat{\beta}_{NS}$ samples are as follows:

$$\hat{\beta}_{S}=\left( k_{O}+{0.5k}_{M}+{0.5k}_{P}+C \right)$$

$$\hat{\beta}_{NS}=\left( k_{O_{1}}+{0.5k}_{M}+{0.5k}_{P}+{0.5k}_{O_{2}}+C \right)$$

The simulations also confirmed the expectations of bias relating to violation of the assumptions and that the difference estimator $\hat{\beta}_{NS}-\hat{\beta}_{S}$ provides an unbiased estimate of sibling indirect genetic effects if all the assumptions hold.

Model 2 – Non-random mating

In Model 2, we extended Model 1 by modifying the variance-covariance matrix of the within-family PGS to reflect assortative mating. This involved increasing the maternal-paternal correlations from 0 to 0.2 and the parent-offspring and sibling correlations from 0.5 to 0.6. Phenotypes were simulated as in Model 1.

a) Parent-offspring trios

$$M P O$$

$$\begin{matrix} 1 & 0.2 & 0.6 \\ 0.2 & 1 & 0.6 \\ 0.6 & 0.6 & 1 \end{matrix}$$

b) Parent-offspring trios

$$M P O_{1} O_{2}$$

$\begin{matrix} 1 & 0.2 \\ 0.2 & 1 \end{matrix}$ $\begin{matrix} 0.6 & 0.6 \\ 0.6 & 0.6 \end{matrix}$

$\begin{matrix} 0.6 & 0.6 \\ 0.6 & 0.6 \end{matrix}$ $\begin{matrix} 1 & 0.6 \\ 0.6 & 1 \end{matrix}$

In Model 2, the OLS regression estimates $\hat{\beta}_{S}$ and $\hat{\beta}_{NS}$ were slightly higher than in Model 1 because of the increased covariance of the PGS within-families. Notably, the difference term $(\hat{\beta}_{NS}$ - $\hat{\beta}_{S}$) was also inflated over expectation suggesting that assortative mating, if not accounted for, could lead to overestimates of sibling indirect genetic effects from the proposed framework.”

We have modified the discussion to note assortative mating as a potential source of bias.

“Further caveats are that our analyses may have been affected by selection bias relating to non-random participation in UK Biobank ^29; 35^, that we did not account for possible indirect genetic effects of half siblings in our analyses as this data was not available and that we assumed random mating with assortative mating a likely source of bias.”

Minor comment: Page 12, methods: The derivation of Cov_ns(G_i,Y) seems to assume that there are only additive (independent allelic) genetic effects. Although this assumption is reasonable, it should be explicitly stated.

We have modified the text as follows to address this point.

Before: “We assume a single autosomal genetic variant, random mating, no confounding between $G_{I}$ and $Y$ and that non-singletons have one sibling each.”

After: “We assume a single autosomal genetic variant, **additive genetic effects only,** random mating, no confounding between $G_{I}$ and $Y$ and that non-singletons have one sibling each.”

Reviewer #2: This paper focuses on a topic that has attracted a lot of attention, i.e. the estimation of indirect effects in within family designs. The paper focuses on a specific type of indirect effects, i.e. indirect effects that originate in siblings rather than parents. It exploits data from both siblings and singletons to estimate such effects and provides a valuable addition to the field.
General comments
The authors justify their approach by a gain in power from including singletons, which seems fairly intuitive. However, because the estimation methods seem different (here test for a difference in association strength) is it worth justifying this point further either by developing the argumentation of why there is a gain in power (compared to which design) or by simulations?

Within-family designs generally have low statistical power because datasets of genotyped families are often small and because the models are statistically inefficient with power requiring heterozygosity within families. For example, only genotypically discordant siblings will contribute statistical power to a within-sibship regression model. In contrast, our proposed approach uses data from unrelated individuals bypassing these two limitations of within-family models. We have made changes to the text to clarify that the gain in power is because the approach uses data from unrelated individuals and not a within-family model.

Before: “However, existing studies that have sampled family members have limited power to estimate sibling effects, even when genotypes of missing first degree relatives are imputed ^12; 13; 15^. Here, we propose an alternative molecular genetics approach for evaluating indirect genetic effects of siblings that uses large samples of unrelated individuals to maximise statistical power.”

After: “However, existing studies that have sampled family members have limited power to estimate sibling effects **because of the paucity of available family data and the statistical inefficiency of within-family models**, even when genotypes of missing first degree relatives are imputed ^12; 13; 15^. Here, we propose an alternative molecular genetics approach for evaluating indirect genetic effects of siblings which **can use large samples of unrelated individuals and so is likely to have higher statistical power than within-family approaches**.”

Is it possible to include in the article the estimates of sibling effects from other designs (e.g. in adopted siblings or any comparable design using polygenic scores?) to verify whether effects are consistent with the approach used by the authors? At present, we have a suggested method but no benchmark either by simulation or by other designs showing whether this approach is reliable.

It is straightforward to qualitatively discuss estimates of sibling effects from other studies in terms of whether the study found evidence or did not find evidence of sibling indirect genetic effects. However, comparing the estimates quantitatively is challenging because the studies are investigating different parameters and use different methods and data with estimates presented on different scales. Furthermore, as far as we are aware, all previous studies have been severely underpowered to detect sibling indirect genetic effects limiting comparability. We have added discussion of parts of two studies relating to sibling indirect genetic effects below.

1) Mendelian imputation of parental genotypes for genome-wide estimation of direct and indirect genetic effects. *biorXiv* A Young et al.

This study used individual level genetic data from families in UK Biobank along with Mendelian imputation to disentangle direct and indirect genetic effects. Here in Supplemental Table 3, they present LD score SNP heritability estimates for sibling IGEs. For educational attainment, this estimate was -0.0478 with a standard error of 0.0756, imprecise. As the authors note, they have limited statistical power to detect sibling IGEs, and this estimate is also on a different scale to our results (although conversion would be possible under certain assumptions).

2) Estimating effects of parents’ cognitive and non-cognitive skills on offspring education using polygenic scores. biorXiv P Demange et al.

This study included several sensitivity analyses to evaluate evidence for indirect genetic effects of siblings using non-cognitive and cognitive PGS which are contained in the Supplemental Tables 8-9.

The first approach used individual level data from pedigrees from the Netherlands Twin Registry to estimate the effects of sibling non-cognitive and cognitive PGS finding minimal evidence of effects. This approach is limited by statistical power because of homozygosity within families and the limited sample size (<10k). The second approach compared differences between monozygotic and dizygotic twins in terms of PGS effects on EA finding minimal evidence of differences. This approach is also limited by sample size and is potentially sensitive to the equal environments assumption. The third approach involved a similar approach to ours looking at PGS associations by number of siblings in UK Biobank in adopted and non-adopted individuals. The main difference is that they only used a small subset of the sample (<6k), limiting their power, instead of the whole sample as we used (~300k) and focused on the trend in the adopted siblings. They observed a weak trend in both the adopted and non-adopted individuals concluding likely confounding on family size. The concerns over confounding by family-size are likely to be overshadowed by the concerns over confounding by adoption status with only ~1% of the cohort reporting being adopted, suggesting that this group may not reflect the average UK Biobank study participant.

Based on the power limitations of previous studies and other limitations with regard to estimating sibling indirect genetic effects, we believe it is difficult to currently directly compare their estimates to our own. We are hopefully that future studies with larger pedigree datasets will allow more useful comparisons.

In response to reviewer 1, we have added additional simulations (including code) and theory to support the proposed framework. For example, we have provided further detail on how higher PGS-phenotype covariance would be expected amongst non-singletons than singletons if there are sibling IGEs. We have also provided expectations on potential biases.

What about sex of the sibling? Does it make a difference whether sibling is same-sex or different sex If the authors interpret the findings as sibling influences on educational decisions, we could imagine that having a same-sex sibling going to uni would make more of a difference?

The reviewer is suggesting that we investigate whether the different sex combinations of siblings (e.g., brother, sister or brother, brother) affect the magnitude of sibling IGEs.

A challenge with investigating this question is that it isn’t binary whether an individual has a same-sex or opposite-sex sibling as individuals could have both and could have multiple of each. In UK Biobank there is questionnaire data on “number of full-brothers” and “number of full-sisters” which were used to derive the total number of siblings. We included a model where we fit a linear interaction term between number of siblings and the PGS on the educational attainment PGS – educational attainment association. A possible analysis could therefore be to replace the single interaction term of number of siblings with two interaction terms for the numbers of same- and opposite-sex siblings and then to compare the coefficients.

A major caveat of this approach is the limited statistical power to detect heterogeneity with the coefficient for the total number of siblings already relatively imprecise: 0.012 (95% C.I. 0.006, 0.018).

As an exploratory analysis, we ran the proposed analysis using the sample of individuals with 5 or fewer siblings. We found no strong evidence of a difference between the same-sex sibling and opposite-sex interaction terms.

Total number of siblings: 0.012 (95% C.I. 0.006, 0.018)

Number of same-sex siblings: 0.011 (95% C.I. 0.003, 0.019)

Number of opposite-sex siblings: 0.013 (95% C.I. 0.001, 0.021)

Given the imprecision of these estimates, we have only presented these results here and not integrated them into the manuscript. We have also added the following to the discussion.

Discussion: “We also did not evaluate whether sibling indirect genetic effects vary by the sex of the index individual and their siblings (e.g. same sex sibling pairs may be more likely to influence one-another).”

Comment on specific sections
Data from twin design etc. are not only usable “in principle”, they can and are used to estimate these effects. It is fair to say that these approaches are sensitive to modelling assumptions and measurement error, but so is the molecular genetic approach. So maybe present this as a complement to triangulate findings rather than an alternative.

We have modified the text as follows to clarify this point.

Before: “In principle, phenotypic data from twins (monozygotic/dizygotic), foster siblings and only children can be used to estimate sibling effects by considering differences in phenotypic variance, but this approach is sensitive to modelling assumptions and measurement error ^8; 9^.”

After: “Phenotypic data from twins (monozygotic/dizygotic), foster siblings and only children can be used to estimate sibling effects by considering differences in phenotypic variance ^8; 9^. A complementary approach is to use molecular genetic data from extended families to estimate indirect genetic effects of parents and siblings ^1; 4; 10; 11^ ^12-14^, which can then inform the effects of parental and sibling phenotypes.”

I know that it is common in the UKB to adjust for 10 PCAs. The Kong paper adjusted for many more + interactions with birth year. Can the authors justify their choice?

In our analyses, we included the first 10 genomic principal components, sex, and age / birth year as covariates.

To investigate possible differences when including additional principal components, we have rerun the regression of the educational attainment PGS on educational attainment in the four subsamples (singletons, non-singletons, firstborns, non-firstborns) including 20 principal components.

We found minimal evidence of differences in both the association estimates and in the singleton difference estimate (below) when including 10 or 20 principal components. These results suggest that adding additional principal components is unlikely to impact our findings or conclusions.

| **Education PGS association estimate** | **10 Principal components** | **20 Principal components** |
| --- | --- | --- |
| Singleton | 0.21 (0.19, 0.23) | 0.21 (0.19, 0.23) |
| Non-singleton | 0.24 (0.23, 0.25) | 0.23 (0.23, 0.24) |
| Singleton difference (%) | 12% (3%, 22%) | 12% (3%, 21%) |
| Firstborn | 0.21 (0.19, 0.23) | 0.21 (0.19, 0.23) |
| Non firstborn | 0.24 (0.23, 0.26) | 0.24 (0.22, 0.26) |

The reviewer also noted that the Kong paper modelled interactions with birth year, which in this instance would be interactions between birth-year and the PGS association. We acknowledged this as a limitation in the discussion “An additional limitation is that (beyond including birth year as a covariate) we did not account for possible generational effects (i.e., effects of the PGS changing over time) which could induce bias in combination with changes in family size over time.”

We investigated whether it is important to model a linear interaction between birth-year and the PGS association. We extended the above analyses by also including a linear interaction term of the PGS multiplied by age in the four subsamples. The P-values for the interaction coefficients in the four subsamples (P = 0.95, P = 0.07, P = 0.30, P = 0.64) suggests that the linear interaction term is unlikely to improve our models.

Based on these results which suggest modest differences when including additional covariates, we have not made changes to the covariates included in our models. However, at the discretion of the reviewers and editors, we would be happy to rerun all analyses with additional principal components.

It is interesting that the authors used the results from a within-sibship GWAS as sensitivity analysis. Please provide estimates using within-sibship GWAS weights in singletons and sibling families in addition to the attenuation. I think that the sentence “However, associations between this PGS and educational attainment in our sample (of unrelated individuals) may still be affected by these sources of association” requires a bit more explanation. The reader may assume that the point of within-sibship GWAS is to obtain SNP weights that are unaffected by these issues even when implemented in singleton samples (e.g. a SNP whose effect is entirely explained by pop strat or indirect effect would be given a weight of 0 and thus not enter the composition of the polygenic score). (Reading the discussion, it appears I misunderstood this first sentence as the authors do use the within-sibship GWAS to exclude these sources of bias as expected. Maybe the sentence can be clarified and developed to avoid misunderstanding. In particular, it may be good to mention more explicitly that the within-sibship GWAS controls for parental indirect effects but does still retain signal from sibling indirect effect. If it did not then we would expect findings to be radically different).

We thank the reviewer for this comment about the within-sibship GWAS sensitivity analysis as this is quite a nuanced point that is difficult to articulate. A PGS based on a within-sibship analysis should provide better estimates of direct genetic effects minimising biases in the score weightings. Considering the three example variants below, using within-sibship weights will remove genetic variant 3 from the PGS, which has no direct genetic effect, and lower the weighting of genetic variant 1, which has both indirect and direct genetic effects.

Genetic variant 1: Direct effect = 0.5, Indirect effect = 0.5.

Within-sibship 0.5, Population 1

Genetic variant 2: Direct effect = 0.5, Indirect effect = 0.

Within-sibship 0.5, Population 0.5

Genetic variant 3: Direct effect = 0, Indirect effect = 0.5.

Within-sibship 0, Population 0.5

The within-sibship PGS will still include genetic variants with indirect effects on educational attainment as long as they also have direct genetic effects, albeit with lower weightings. The lower weighting will not remove the covariance between the genetic variant and the phenotype induced by parental indirect genetic effects. Therefore, the regression of the within-sibship PGS on educational attainment in a sample of unrelated individuals may still capture indirect genetic effects and other non-direct sources of genetic association.

We have made the following changes to the results and discussion to clarify this point:

Results

Before: “However, associations between this PGS and educational attainment in our sample (of unrelated individuals) may still be affected by these sources of association.”

After: “However, associations between this PGS and educational attainment in our sample (of unrelated individuals) may still be affected by these sources of association. **This is because genetic variants in the within-sibship PGS with direct genetic effects on educational attainment may also have non-direct genetic effects which are not controlled for in models that don’t account for parental genotypes.**”

Discussion

Before: “This suggests that our results are unlikely to be explained by effects of population stratification, assortative mating and indirect genetic effects on the PGS. However, these mechanisms could still affect the association between the PGS and educational attainment differently between singletons and non-singletons.”

After: “This suggests that our results are unlikely to be explained by effects of population stratification, assortative mating and indirect genetic effects on the PGS **weightings**. However, these mechanisms could still affect the association between the PGS and educational attainment differently between singletons and non-singletons because analyses in unrelated individuals do not account for variance in parental genotypes.”

Before: “As a sensitivity analysis, we performed analyses using an educational attainment PGS weighted by within-sibship GWAS estimates ^20^ and found consistent evidence that PGS association estimates are larger in non-singletons.”

After: “As a sensitivity analysis, we performed analyses using an educational attainment PGS weighted by within-sibship GWAS estimates ^20^ which are robust against population stratification, assortative mating and indirect genetic effects of parents but not indirect genetic effects of siblings. Here we found consistent evidence that PGS association estimates are larger in non-singletons.”

As requested, we have added the within-sibship GWAS weight estimates to Supplementary Table 1. The attenuated PGS association estimates when using the within-sibship weightings are expected because of the lower statistical power of the within-sibship GWAS compared to the discovery population-based GWAS.

**Supplementary Table 1** PGS association estimates for singletons and non-singletons.

| **Outcome** | **Non-singletons**  **(N = 328,549)** | **Singletons**  **(N = 50,143)** | **Singleton difference % (95% C.I.)** | **Heterogeneity P-value** |
| --- | --- | --- | --- | --- |
| Height:  Increase (cm) per SD increase in height PGS | 2.32 (2.30, 2.34) | 2.32 (2.27, 2.38) | 0% (-3%, 2%) | 0.86 |
| BMI:  Increase (kg/m2) per SD increase in BMI PGS | 0.73 (0.72, 0.75) | 0.70 (0.66, 0.74) | 5% (-1%, 11%) | 0.13 |
| Education:  Increase (years) per SD increase in education PGS | 0.24 (0.23, 0.25) | 0.21 (0.19, 0.23) | 12% (3%, 22%) | 0.009 |
| Education analysis  using within-sibship GWAS weights | 0.18 (0.17, 0.18) | 0.16 (0.14, 0.18) | 11% (-1%, 23%) | 0.08 |

PGS associations and birth order
Sentence: “suggesting that the larger PGS association
in non-singletons is due to individuals with older siblings, rather than firstborns” makes sense but Figure 3 shows that PGS association is higher even in firstborns compared to non-singletons, so technically both would contribute to larger association in the larger non singleton sample. Maybe worth considering/discussing why firstborn and non-firstborn associations are higher than in the rest of the sample (e.g; selection effect) ?
“we found limited evidence for an interaction (P = 0.24)” I know that P values are not the best indicator, but this is what the authors are using here, and pvalue of 0.24 is “no evidence” rather than “limited”.

Apologies there was human error in the creation of Figure 3 with it containing incorrect estimates. Thank you for drawing attention to this mistake. The correct estimates which were correctly contained in Supplemental Tables 1 and 2 are now displayed in Figure 3.

Before:

After:

Regarding “I know that P values are not the best indicator, but this is what the authors are using here, and pvalue of 0.24 is “no evidence” rather than “limited”.” We prefer to interpret P-values on a scale of continuous evidence rather than in terms of thresholds (REF: Sifting the evidence—what's wrong with significance tests?, BMJ 2001). However, we agree with the reviewer that “limited” is potentially confusing and so we have changed “limited evidence” to “no strong evidence” throughout.

The whole approach relies on comparing the strengths of association in families with singletons vs non-singletons. And the difference is attributed to indirect genetic effects. This assumes that those families do not differ by any other characteristics, which may be a stretch. Same applies to the finding that the association is non-linear and different in families with 6+ children. There must be plenty of other factors that distinguish fairly rare families nowadays (6+ children) from others (e.g. religion, income available for each child). To what extent do these possible systematic differences affect the findings? The authors “lower investment or other confounders” in passing but not how these could affect estimates. (this is discussed more in the discussion section but may be worth flagging earlier in the intro or the results that this is an issue discussed at more lengths in methods and discussions).

The reviewer is correct that there are group-level differences between families of different sizes which we investigated empirically in UK Biobank. However, as we discuss in the methods section, group-level differences will not necessarily confound genetic association estimates. Germline genotype is fixed from birth and so there are a limited number of possible confounders of genetic associations with population stratification being the most notable confounder. In response to reviewer 3, we added in a comparison of birth coordinates by family-size to investigate differences in ancestry by family size. As the reviewer suggested, we have made the following additions to the introduction and results sections to make this clearer.

Introduction

Before: “If other sources of genetic association are consistent between singletons and non-singletons, then differences between the singleton and non-singleton genetic association estimates could indicate indirect genetic effects of siblings (**Figure 1**).”

After: “If other sources of genetic association are consistent between singletons and non-singletons, then differences between the singleton and non-singleton genetic association estimates could indicate indirect genetic effects of siblings. **Potential group-level differences between singletons and non-singletons are unlikely to confound genetic associations unless the factor can influence genotype (e.g. ancestry)** (**Figure 1**).”

Results

Before: “These findings illustrate differences between singletons and non-singletons, which could relate to parental differences (e.g. education) but also birth order effects or factors influencing study participation.”

After: “These findings illustrate differences between singletons and non-singletons, which could relate to parental differences (e.g. education) but also birth order effects or factors influencing study participation. **However, we note that group-level differences by family size are unlikely to confound our genetic association analyses with the exception of ancestral differences and that some of the observed differences are relatively modest (e.g. 5.2 km difference in birth coordinates).**”

Looking at Figure 4 makes me question the decision to fit a linear effect after removing 6 or more siblings, which seems post hoc and arbitrary. There seems to be a flattening already from 3 siblings. I think it is worth first fitting a model including both a quadratic and a linear term for number of siblings to check whether there is an overall significant pattern and only conduct linear analyses as follow-up analysis, restricting those analyses to the linear part of the fitted curve (or better, use the linear term in the quadratic model to estimate interaction with PGS). My intuition would be that such a linear term accounting for the quadratic trend should be larger than the one that the authors estimated).

As the reviewer suggested, we have added in the following quadratic model to investigate a non-linear relationship between number of siblings and the PGS on educational attainment.

Education ~ PGS + Siblings + GRS*Siblings + Siblings^2 + GRS*Siblings^2 + Covariates

Applying this model to the full sample (i.e., not removing those with 6 or more siblings), the linear GRS-number of siblings interaction term was estimated to be 0.017 (95% C.I. 0.008, 0.025; P = 0.0001) with the quadratic interaction term estimated to be -0.002 (95% C.I. -0.001, -0.003; P = 0.0003). These findings are consistent with a non-linear relationship between the number of siblings and the PGS association with educational attainment.

We have integrated this analysis into the manuscript alongside the “removing individuals with 6 or more siblings” analysis which we have clarified is a linear analysis with outliers removed.

Results

Before: “After removing these individuals (4.9% of the sample) and repeating analyses in individuals with 5 or fewer siblings we found evidence for a linear relationship; each additional sibling corresponded to an increase of 0.012 in the PGS association estimate (95% C.I. 0.006, 0.018; P = 4.3x10^-5^). Under assumptions discussed in the **Methods**, this estimate can be scaled (multiplied by two) to provide an estimate of sibling indirect genetic effects; a 1 SD increase in the educational attainment PGS of a sibling increases an index individual’s years in schooling by 0.025 years (0.013, 0.036). For comparison, this estimate is 11% (95% C.I. 5%, 16%) of the magnitude of the PGS association estimate in the full sample (0.23 years, 95% C.I. 0.23, 0.24).”

After: “We evaluated whether there is a non-linear relationship between number of siblings and the PGS associations by applying a quadratic model including the square of the number of siblings and a quadratic interaction term. This model provided evidence of a non-linear relationship with the linear interaction estimate indicating a 0.017 increase in PGS association estimate per each additional sibling (95% C.I. 0.008, 0.025; P = 0.0001) in the opposite direction to the quadratic interaction estimate which indicated a 0.002 decrease in PGS association estimate per each unit increase in the square of the number of siblings (95% C.U. -0.001, -0.003; P = 0.0003).

We also repeated the linear analysis after removing outlying individuals with extreme values, considering individuals with 6 or more siblings as outliers based on an outlier threshold of 5% because this group corresponded to 4.9% of the total sample. In this sample of individuals with 5 or fewer siblings we found evidence for a linear relationship; each additional sibling corresponded to an increase of 0.012 in the PGS association estimate (95% C.I. 0.006, 0.018; P = 4.3x10^-5^). Under assumptions discussed in the **Methods**, this estimate can be scaled (multiplied by two) to provide an estimate of sibling indirect genetic effects; a 1 SD increase in the educational attainment PGS of a sibling increases an index individual’s years in schooling by 0.025 years (0.013, 0.036). For comparison, this estimate is 11% (95% C.I. 5%, 16%) of the magnitude of the PGS association estimate in the full sample (0.23 years, 95% C.I. 0.23, 0.24).”

One question that may not be pertinent. The analysis seems to suggest that non singletons and non-firstborns (with not too many siblings) benefit from education-increasing indirect genetic effects via their sibling. How does it translate at the phenotypic level? Does it only make a difference in variances or also a difference of means (i.e. having a sibling helps with schooling). In an MR framework this association could be consistent with a causal effect of sibling education on the focal child’s education. I guess the effect would only increase the focal’s child education at the phenotypic level if the sibling had a higher polygenic load for education, and would decrease it if they had a lower polygenic load, leading to no expected differences in average between singletons and non-singletons.

The theory and analysis doesn’t necessarily suggest that non-singletons will benefit as sibling indirect genetic effects could affect a phenotype in both directions. It suggests that non-singletons and non-firstborns may converge to the phenotypes of older siblings. For example, if the older sibling has a higher education PGS then on average the sibling IGE would increase the education of the younger sibling but if the older sibling has a low education PGS, then the sibling IGE would decrease the education of the younger sibling. Therefore, differences in mean phenotype would not be expected between singletons and non-singletons because of indirect genetic effects of siblings. Non-singletons would have an additional source of variation so might be expected to have higher phenotypic variance, but this is complex as is discussed in the response to the reviewer’s next comment.

Is it possible to provide the descriptives and formally test the differences in both variances and means as well as discuss what should we expect and what we observe at the phenotypic level. (Reading the rest of the results, I see that the authors provided some of the descriptives between singleton/nonsingletons and firstborns and nonfirstborns, including mean differences in education. I think it would be worth to also formally test for differences of variance in education and discuss these findings in a systematic way, i.e. spell out earlier in the text rather than in the discussion whether we expect differences in means and variances or not, and if yes, in which direction. And discuss whether findings are consistent or in contradiction to what would be expected under the indirect effect model?

As discussed in the response to the reviewer’s preceding comment, sibling indirect genetic effects as modelled in our framework would not be expected to lead to differences in phenotype means between non-singletons and singletons but would be expected to increase phenotypic variance as an additional source of variance. As the reviewer notes, we provided estimates of the means and variances of different phenotypes in non-singletons and singletons in Supplementary Table 3. The variance of educational attainment was similar between non-singletons and singletons.

However, the challenge of interpreting this analysis is that there are many group-level differences between singletons and non-singletons which could potentially lead to differences in variance much larger than any from sibling indirect genetic effects. For example, the mean birth year is 3 years earlier in singletons and a higher proportion of singletons were male which could both impact phenotypic variance. In contrast, these group-level differences are less likely to confound genetic analyses because very few factors can influence germline genotype. We could potentially compare variances after residualising on potentially confounding factors in the variance analysis such as birth year and sex, but the difficulties are that it is unclear which measured factors to residualise on and that there may be unmeasured factors influencing variance differences between singletons and non-singletons.

Methods
“Indirect genetic effects of the maternal (), paternal ( ) and sibling ()
genotype on via the shared environment. These effects are captured by [ , ]”
Maybe “partially” captured? Cov[G1,Y] only captures indirect effects of transmitted rather than untransmitted parental alleles (i.e. )?

We have modified this sentence as suggested from “these effects are captured” to “these effects are partially captured”.

Linked to that, in the second equation we get 0.5Kns,m from KmGm in the first equation. Readers less familiar with these models may benefit from an explicit demonstration of this (i.e; comes from cov(G1,GM) being 0.5) as well as a more detailed derivation (maybe as supplementary). Also, not sure I get why *Var[G1] applies to the whole expression in brackets rather than only the first term (i.e. cov(G1,k1G1) = k1Var[G1]), which I could understand with the full derivation. This is important as the expression for the beta depends on Var[G1] applying to the full expression (although not sure it matters practically as variances of polygenic scores are unity).

We have added more detail to the Supplement on the covariance derivation as detailed below. It is our understanding that cor(GI, GM) = 0.5 so cov(GI, GM) = 0.5 Var(GI).

“We define a phenotype $Y$ as a function of the index individual ($G_{I}$), maternal ($G_{M}$), paternal ($G_{P}$) and sibling ($G_{S}$) genotypes.

$$Y = k_{I}G_{I} + k_{M}G_{M}+k_{P}G_{P}+k_{S}G_{S}+\in$$

where$k_{I,M,P,S}$are the (direct/indirect) effects of $G_{I,M,P,S}$ on $Y$

Our aim is to derive the covariance between $G_{I}$ and $Y$ in singletons ${(Cov}_{S}[G_{I}, Y] )$and non-singletons ${(Cov}_{NS}[G_{I}, Y] )$. Here, we derive ${Cov}_{NS}[G_{I}, Y]$.

First, we consider the general derivation for covariance in terms of expectations.

(1) $Cov[X, Y] = E[XY] - (E[X]E[Y])$

From (1) we note that:

$${Cov}_{NS}[G_{I}, Y] = E[G_{I}Y] - (E[G_{I}]E[Y])$$

We can expand $E\left[ G_{I}Y \right]$ as follows:

$$E\left[ G_{I}Y \right]= E\left[ k_{I}{G_{I}}^{2}+k_{M}G_{M}G_{I}+k_{P}G_{P}G_{I}+k_{S}G_{S}G_{I} \right]$$

$$= k_{I}E[{G_{I}}^{2}] + k_{M}E\left[ G_{M}G_{I} \right]+k_{P}E\left[ G_{P}G_{I} \right]+k_{S}E[G_{S}G_{I}]$$

From (1) it follows that:

$E[{G_{I}}^{2}]=E$[$G_{I}]^{2}+{Cov}_{NS}[G_{I}, G_{I}]$

$$=E[G_{I}]^{2} + Var[G_{I}]$$

From (1) and the correlation of 0.5 between $G_{I}$ and $G_{M,P,S}$ it follows that:

$E[G_{M}G_{I}$] $=E\left[ G_{P}G_{I} \right]=E\left[ G_{S}G_{I} \right]$

$$= E\left[ G_{M} \right]E\left[ G_{I} \right]+{Cov}_{NS}[G_{M}, G_{I}]$$

$$=E[G_{I}]^{2} + 0.5Var[G_{I}]$$

Therefore, we can simplify $E\left[ G_{I}Y \right]$ to the following:

(2) $E\left[ G_{I}Y \right]$ =$\left( k_{I}{+0.5k}_{M}+{0.5k}_{P}+{0.5k}_{S} \right)Var[G_{I}]$ + $\left( k_{I}{+k}_{M}+k_{P}+k_{S} \right)E[G_{I}]^{2}$

We can derive $E\left[ Y \right]$ as a function of $k_{I,M,P,S}$ and ${E[G}_{1}]$:

$$E\left[ Y \right]=\left( k_{I}+k_{M}+k_{P}+k_{S} \right)E[G_{I}]$$

It then follows that:

$$\left( 3 \right)E[G_{I}]E[Y])=\left( k_{I}+k_{M}+k_{P}+k_{S} \right)E[G_{I}]^{2}$$

Finally, from (1), (2) and (3) we can simplify ${Cov}_{NS}\left[ G_{I}, Y \right]$ to:

${Cov}_{NS}\left[ G_{I}, Y \right]= \left( k_{I}+{0.5k}_{M}+{0.5k}_{P}+{0.5k}_{S} \right)Var[G_{I}]$”

“We constructed PGS for height, BMI and educational attainment by LD
Clumping (P < 1.0x10-5, r2 < 0.001, clumping distance = 10000 kb).” This is a fairly basic way of computing the polygenic score. Why choose this p-value, any justification? Why not choose a more advanced method to account for LD like LDpred? This would have the benefit of increasing the accuracy of polygenic scores.

The p-value threshold of 1.0x10^-5^ was chosen as a compromise in terms of statistical power and interpretability of the PGS between the more conservative genome-wide significance threshold 5x10^-8^ and more liberal approaches such as including all markers genome-wide. The genome-wide significance threshold PGS would have the advantage of including very strongly associated markers that are likely to be genuine associations but would have limited statistical power. A score including all markers would have the advantage of higher statistical power but would likely include many spuriously associated markers and so be less interpretable in terms of the phenotypes and potentially more susceptible to potential biases such as population stratification.

The goal of this study was to use PGS to investigate potential indirect genetic effects of siblings rather than to maximise prediction in terms of variance explained where one would likely want to include more markers. We believe that our approach of selecting relatively strongly associated independent markers (based on strict LD thresholds) is sufficient for this aetiological analysis. The simplicity of the approach has advantages in that the results are easy to interpret and that we made few assumptions about genetic architecture or LD.

It seems unlikely that a more complex PGS approach would affect our findings or conclusions. If the reviewer is unconvinced by our response, we are happy to add in a sensitivity analysis using LD Pred or another PGS approach such as megaPRS.

Reviewer #3: In this paper, the authors use a creative design to try and estimate the magnitude of the sibling indirect effects in a PGS analysis for educational attainment, BMI, and height. Namely, they estimate the association of the PGS with its corresponding outcome in four subsamples of the UK Biobank: (a) those who report having no full siblings, (b) those who report having at least one sibling, (c) those who report having only younger siblings, and (d) those who report having at least one older sibling. Under the assumption that the direct and indirect genetic effects are the same across these different groups, a comparison of the PGS associations between these groups might shed light on whether a person's outcomes may be affected by the PGS of their sibling.

While I liked the idea behind the design of this study, I find myself very concerned about the limitations of this approach. I really appreciated that many of these limitations are brought up in a careful way in the discussion and methods section, though a bit more discussion on how important these limitations are would be useful. I describe these concerns in more detail below:

1. The authors show in the methods section how a key assumption of their design is that the direct genetic effects and indirect genetic effects of parents is the same in the different groups they consider. They state that one piece of evidence in support of their assumptions would be if the groups were similar on other observable characteristics. However, when they make comparisons across these different groups, they find significant differences in means on a number of variables. So how likely is it that their key assumption holds? One thing they may consider is to take the variables where they detect significant differences (e.g., men vs women) and test whether the PGS has a different coefficient when you divide the same that way. They may also consider other variables that are fixed early in life like birth coordinates.

As the reviewer notes, we found evidence of group-level differences between singletons and non-singletons for age, sex, and several phenotypes. However, as we note in the methods, these group-level differences may not necessarily distort the different sources of genetic association. Group-level differences will only induce confounding bias if they influence both genotype and the outcome which is not possible for sex, age, and many other phenotypes which cannot plausibly influence germline genotype. As we discuss, interactions or non-linear effects of the PGS on the outcome could combine with group-level phenotypic differences to induce some heterogeneity in genetic associations between singletons and non-singletons.

We agree that birth coordinates are something that would be useful to compare between singletons and non-singletons, as population stratification could be a plausible confounder in our analyses. Therefore, we have added in birth coordinate comparisons between singletons, non-singletons, firstborns and non-firstborns which are detailed in Supplementary Tables 3-5. We found statistical evidence of differences between singletons and non-singletons in the mean birth coordinates but these differences were relatively modest (3.5 km and 5.2km compared to standard deviations of 157.4 km and 164.6 km respectively). We have integrated these results into the results.

Results

“We compared the sex, age, height, BMI, measured educational attainment and educational attainment PGS of singletons and non-singletons. Singletons were more likely to be male (+1.0%; 95% C.I. 0.6%, 1.5%), older (+2.6 years; 95% C.I. 2.6, 2.7) and were born further south (5.2 km; 95% C.I. 3.6, 6.8) and east (3.5km; 95% C.I. 2.7, 4.3).”

The reviewer suggested testing differences in PGS associations on factors that differ between non-singletons and singletons such as by sex to inform potential bias. Notably this has already been looked into by several groups (e.g. *“Variable prediction accuracy of polygenic scores within an ancestry group” eLife 2020*) and the results suggest that there are differences in PGS associations across different covariates and groups.

However, we note that the group-level differences we observe between singletons and non-singletons are modest and so this approach seems unlikely to uncover major bias. For example, if the PGS association differs between men and women by X, then 46.8% of singletons are male compared to 45.8% of non-singletons, so we would expect the sex differences to contribute a difference of 0.01 X. Furthermore, the total contribution of group-differences would be unclear as there may be unmeasured differences modifying the association in either direction. Our preference is therefore to be up front about the limitations of the approach and encourage triangulation of evidence with pedigree-based methods. We have modified the text to note that group-level differences are unlikely to confound the genetic association analyses and that some of the observed differences are modest.

Results

Before: “These findings illustrate differences between singletons and non-singletons, which could relate to parental differences (e.g. education) but also birth order effects or factors influencing study participation.”

After “These findings illustrate differences between singletons and non-singletons, which could relate to parental differences (e.g. education) but also birth order effects or factors influencing study participation. However, we note that group-level differences by family size are unlikely to confound our genetic association analyses with the exception of ancestral differences and that some of the observed differences are relatively modest (e.g. 5.2 km difference in birth coordinates).”

2. The PGS the authors use are based on population-level GWAS that are blind to the family size of the individuals in the sample. If there is GxE on family size and the GWAS sample has more individuals with at least one sibling, then we would expect the PGS to be more predictive in a sample of people with siblings, wouldn't we. Given that most families have at least two children, it seems sensible that this could be a problem.

The reviewer is suggesting that a PRS derived from the discovery population GWAS could be more strongly associated with educational attainment in non-singletons than singletons if there are GxE interactions on family size because most people in the discovery GWAS will have singletons.

We believe this suggestion is consistent with the assumption of no differences in direct genetic effects between singletons and non-singletons which we noted as a core assumption of the framework. We also note that we ran a sensitivity analysis using a PGS weighted by a within-sibship GWAS (i.e. no singletons) and noticed minimal evidence of differences in the primary analysis.

3. I was uncomfortable with the author's decision to drop estimates that correspond to families with 6 or more siblings. Once you acknowledge that environmental factors (like parental investment) may be influencing the patterns seen in the results, why couldn't it be that all the results are being driven by these factors?

We removed the families with 6 or more siblings in a single analysis estimating a linear relationship between number of siblings and educational attainment PGS associations. In all other analyses, such as the comparison between the singleton and non-singleton PGS association estimates these families were not removed.

For the linear relationship analysis, there are three challenges with including individuals from larger families with 7 or more children. First, the data on number of siblings is self-report and so the reliability of the data from individuals reporting very high numbers of siblings is unclear. From the UK Biobank data showcase, participants were asked to confirm if self-reporting more than 10 brothers or 10 sisters, the two variables which we summed ([: Data-Field 1883 (ox.ac.uk)](https://biobank.ndph.ox.ac.uk/showcase/field.cgi?id=1883)). Extreme spurious outliers could be problematic for the linear model. Second, UK Biobank is known to be a highly non-random sample with study participants being much more highly educated on average than the general population. Family size is inversely associated with education-level because more education generally leads to having children later, and so study participants with very large families are likely to be atypical for the study population, a potential source of selection bias. Third, a linear relationship with each additional sibling may be plausible in smaller families (e.g., 2 to 3 siblings) but is less implausible in much larger families.

In analyses of self-report epidemiological data, it is common practice to remove outlying individuals reporting extreme values. Here, we chose the exclusion threshold of 6 or more siblings because this group corresponded to just under 5% of the sample.

As the reviewer notes and as we discuss extensively in the manuscript, family size is likely to be associated with environmental factors. However, we believe that when modelling a linear relationship and considering potential confounding, there is a distinction between comparing groups close to the mean of the distribution (e.g., 1 sibling versus 2 siblings) with groups increasingly far from the mean (e.g., 7 siblings versus 8 siblings) because of the potential increased misclassification and selection biases on these much larger families.

In response to comments from reviewer 2, we have also added in a non-linear model for this analysis. We have made the following changes to the text:

Results

Before: “However, this sample included very large families where the association estimate may be affected by lower parental investment or other confounding factors. Indeed, we found that the PGS association estimate from 18,746 individuals self-reporting having 6 or more siblings was actually 24% smaller than the estimate from individuals with 5 or fewer siblings (95% C.I. 11%, 38%, P = 5.2x10^-4^) (**Figure 4**).”

After: “However, this sample included very large families where **data could be more susceptible to misclassification error and** the association estimates may be affected by lower parental investment or other confounding factors. Indeed, we found that the PGS association estimate from 18,746 individuals self-reporting having 6 or more siblings was 24% smaller than the estimate from individuals with 5 or fewer siblings (95% C.I. 11%, 38%, P = 5.2x10^-4^) (**Figure 4**).”

Before: “After removing these individuals (4.9% of the sample) and repeating analyses in individuals with 5 or fewer siblings we found evidence for a linear relationship; each additional sibling corresponded to an increase of 0.012 in the PGS association estimate (95% C.I. 0.006, 0.018; P = 4.3x10^-5^).”

After: “We evaluated whether there is a non-linear relationship between number of siblings and the PGS associations by applying a quadratic model including the square of the number of siblings and a quadratic interaction term. This model provided evidence of a non-linear relationship with the linear interaction estimate indicating a 0.017 increase in PGS association estimate per each additional sibling (95% C.I. 0.008, 0.025; P = 0.0001) in the opposite direction to the quadratic interaction estimate which indicated a 0.002 decrease in PGS association estimate per each unit increase in the square of the number of siblings (95% C.U. -0.001, -0.003; P = 0.0003).

We also repeated the linear analysis after removing outlying individuals with extreme values, considering individuals with 6 or more siblings as outliers based on an outlier threshold of 5% because this group corresponded to 4.9% of the total sample. In this sample of individuals with 5 or fewer siblings we found evidence for a linear relationship; each additional sibling corresponded to an increase of 0.012 in the PGS association estimate (95% C.I. 0.006, 0.018; P = 4.3x10^-5^). Under assumptions discussed in the **Methods**, this estimate can be scaled (multiplied by two) to provide an estimate of sibling indirect genetic effects; a 1 SD increase in the educational attainment PGS of a sibling increases an index individual’s years in schooling by 0.025 years (0.013, 0.036). For comparison, this estimate is 11% (95% C.I. 5%, 16%) of the magnitude of the PGS association estimate in the full sample (0.23 years, 95% C.I. 0.23, 0.24).”

4. In the methods section, they work out the math to infer how large the indirect effect from siblings under the assumptions of their method. Eyeballing figure 3, the difference between singletons and non-firstborns is about .07, suggesting the coefficient on the sibling PGS would be .14, almost as big as the association in singletons. Is this plausible?

Apologies there was human error in the creation of Figure 3 with it containing incorrect estimates. Thank you for drawing attention to this mistake. The correct estimates which were correctly contained in Supplemental Tables 1 and 2 are now displayed in Figure 3.

Before:

After:

The difference between singleton and the non-first-born estimates is 0.03, suggesting the coefficient would be 0.06, which is < 30% of the singleton PGS estimate.

5. Overall, I'd bring the assumptions and limitations much more front and center and expand the discussion of whether those assumptions are justified. And if they aren't justified, how large do you anticipate the biases would be as a result of the confounds.

Currently most of our discussion about the assumptions and limitations of the proposed framework is contained in the methods section. We agree with the reviewers that it would be better to describe these limitations in earlier sections. Therefore, we have made the following additions to the abstract and introduction.

Abstract

Before: “Indirect genetic effects of siblings increase (or decrease) the covariance between genetic variation and a phenotype. It follows that differences in genetic association estimates between singletons and non-singletons could indicate indirect genetic effects of siblings.”

After: “Indirect genetic effects of siblings increase (or decrease) the covariance between genetic variation and a phenotype. It follows that differences in genetic association estimates between singletons and non-singletons could indicate indirect genetic effects of siblings **if there is no heterogeneity in other sources of genetic association between singletons and non-singletons**.”

Introduction

Before: “Extending this intuition, we note that indirect genetic effects of siblings will not impact genetic association estimates from singletons (individuals without siblings). If other sources of genetic association are consistent between singletons and non-singletons, then differences between the singleton and non-singleton genetic association estimates could indicate indirect genetic effects of siblings (**Figure 1**).”

After: “Extending this intuition, we note that indirect genetic effects of siblings will not impact genetic association estimates from singletons (individuals without siblings). **Therefore, differences between singleton and non-singleton genetic association estimates could indicate indirect genetic effects of siblings if there is no heterogeneity in the other sources of genetic association between singletons and non-singletons (Figure 1).”**

In response to comments from another reviewer, we have also included additional theory and simulations exploring how sensitive the estimates are to assumptions and biases detailed above.
