# Supplementary material for: Evaluating indirect genetic effects of siblings using singletons
Source: PLoS Genet. 2022 Jul 7;18(7):e1010247. doi: 10.1371/journal.pgen.1010247 (PMC9262210; doi:10.1371/journal.pgen.1010247)
Supplement: Attachment — Submitted filename: SiblingIGE_response_May2022 (1).docx [file pgen.1010247.s005.docx]

Thank you very much for submitting your Research Article entitled 'Evaluating indirect genetic effects of siblings using singletons' to PLOS Genetics.

The manuscript was fully evaluated at the editorial level and by independent peer reviewers. The reviewers appreciated your improvements and revisions, but identified some remaining concerns that we ask you address in a revised manuscript. Specifically, as mentioned by one reviewer, a sentence in the discussion and a short supplementary note describing how your results compare to existing estimates using more direct methods (something along the lines of what you wrote in the response) would be valuable to readers.

We therefore ask you to modify the manuscript according to the review recommendations.

In addition we ask that you:
1) Provide a detailed list of your responses to the review comments and a description of the changes you have made in the manuscript.
2) Upload a Striking Image with a corresponding caption to accompany your manuscript if one is available (either a new image or an existing one from within your manuscript). If this image is judged to be suitable, it may be featured on our website. Images should ideally be high resolution, eye-catching, single panel square images. For examples, please browse our [archive](https://journals.plos.org/plosgenetics/volume). If your image is from someone other than yourself, please ensure that the artist has read and agreed to the terms and conditions of the Creative Commons Attribution License. Note: we cannot publish copyrighted images.

We thank the editors and reviewers for their helpful feedback on our manuscript. We have responded below to the reviewer comments. We hope that our revised manuscript is better suited for publication in PLOS Genetics.

Reviewer #3: I thank the authors for their clarifying comments I am substantially less concerned than I was before.

The point about the units of educational attainment is well taken. I'd note, however, that the PGS in Kong et al. is substantially more predictive than the PGS used in this paper. (I believe the implied R2 in this paper is roughly 1% while it is over 5% in Kong et al.) That said, if you assess the magnitude of the indirect sibling effect as a fraction of the population effect, I calculate a 95% confidence interval of (-11%,10%) for Kong et al. and (5%,15%), so they remain largely consistent even when accounting for differences in predictive power of the PGS.

If the editor agrees, I think a sentence in the discussion and a short supplemental note of how your estimates compare to more noisy estimates of indirect genetic effects which use sibling data would be valuable to readers.

Thanks to the reviewer for their helpful comments and for pointing out how differences in the PGS can complicate direct comparisons. We have added a paragraph to the discussion as well as a supplementary note describing the comparison. Because of the methodological (study population, variants included in the PGS) differences between our study and Kong et al, we noted that the direct comparison of estimates between the two studies has limitations.

**Discussion:**

“In contrast to our findings, previous studies using family-based approaches have reported limited evidence of sibling IGEs. This could have been because of differences in statistical power or because of genuine heterogeneity. We compared our sibling IGE estimates to the estimates from one of these manuscripts (Kong et al ^13^) and found limited evidence of heterogeneity. Our sibling IGE estimate, which was more precisely estimated, was consistent with the estimate and 95% confidence interval from Kong et al ^13^ (**Supplementary Material**). This suggests that the differences in conclusions are likely to relate to differences in statistical power.”

**Supplementary note:**

Comparison of Howe et al sibling IGE estimates with Kong et al

In this manuscript, we report evidence of sibling IGEs using singleton status data in unrelated individuals. In a recent preprint, Kong et al reported limited evidence of sibling IGEs using a family-based approach.

Kong, A., Benonisdottir, S., & Young, A. I. (2020). Family analysis with Mendelian imputations. BioRxiv.

To evaluate whether differences in conclusions relate to statistical power or to genuine heterogeneity, we compared the estimates. The two analyses used different (overlapping) datasets from the UK Biobank and distinct methodologies. For example, Kong et al used an educational attainment PGS including a larger number of variants (and so is more predictive) than the PGS that we used suggesting that caution is required when interpreting a direct comparison.

Kong et al estimated that a SD increase in the sibling’s educational attainment PGS corresponds to a -0.001 SD unit change in the index individual’s educational attainment (95% C.I. -0.026, 0.024). We estimated that a SD increase in a sibling’s PGS corresponds to a 0.025 (0.013, 0.036) year increase in the index individual’s educational attainment.

Kong et al presented their estimates in standard deviation units while we present our estimates in years in educational attainment. Therefore, considering that the standard deviation of educational attainment is 2.3 years (after adjusting for sex and birth year), we rescaled the Kong et al estimates to be expressed in terms of years of educational attainment and so more directly comparable to our estimates. The scaled Kong et al estimate is that a SD increase in the sibling’s educational attainment PGS corresponds to a -0.0023 year (95% C.I. -0.061, 0.057) change in the index individual’s educational attainment. We also scaled the overall PGS association estimate from Kong et al to years of education and compared the ratio of the sibling IGE estimates over the overall PGS association.

|  | Howe et al  *Estimate (95% C.I.)* | Kong et al  *Estimate (95% C.I.)* |
| --- | --- | --- |
| Sibling PGS to index individual educational attainment  (years of schooling) | 0.025 (0.013, 0.036) | -0.002 (-0.061, 0.057) |
| Index PGS to index individual educational attainment  (years of schooling) | 0.23 | 0.55 |
| Sibling PGS association divided by index PGS association  (%) | 11% (6%, 16%) | 0% (-11%, 10%) |

There was limited statistical evidence of heterogeneity between the sibling IGE estimates from the two studies. The ratios of the sibling PGS estimate over the overall PGS association estimate were also broadly consistent between the two studies. This suggests that differences in statistical power are likely to explain the differences in conclusions between our work and that of Kong et al.
